# Efficacy of Intellect’s self-guided anxiety and worry mobile health programme: A randomized controlled trial with an active control and a 2-week follow-up

**DOI:** 10.1371/journal.pdig.0000095

**Published:** 2023-05-24

**Authors:** Feodora Roxanne Kosasih, Vanessa Tan Sing Yee, Sean Han Yang Toh, Oliver Sündermann

**Affiliations:** 1 Research Department, Intellect Co Pte Ltd, Singapore; 2 Department of Psychology, National University of Singapore; University of Bayreuth: Universitat Bayreuth, GERMANY

## Abstract

Digital self-guided mobile health [mHealth] applications are cost-effective, accessible, and well-suited to improve mental health at scale. This randomized controlled trial [RCT] evaluated the efficacy of a recently developed mHealth programme based on cognitive-behavioral therapy [CBT] principles in improving worry and anxiety. We also examined psychological mindedness [PM] as a mediator by which app engagement is thought to improve outcomes. The Intervention group completed a 2-week “Anxiety and Worry” programme with daily CBT-informed activities, while the active waitlist-control completed a matched 2-week mHealth programme on procrastination. Participants filled out the Generalized Anxiety Disorder [GAD-7], Patient Health Questionnaire [PHQ-9], and Psychological Mindedness Scale [PMS] at baseline, post-intervention, and 2-week follow-up. App engagement was measured at post-intervention only. Contrary to prediction, the Intervention group did not perform better than the Active Control group; both groups showed significant improvements on anxiety and depressive symptoms from baseline to follow-up. From post-intervention to follow-up, only the Intervention group showed further improvements for anxiety symptoms. Higher engagement with the mHealth app predicted lower anxiety and depressive symptoms at follow-up, and this relationship was fully mediated by psychological mindedness. This study provides evidence that [a] engaging in a CBT mHealth programme can reduce anxiety and worry, and [b] Psychological mindedness is a potential pathway by which engaging with a mHealth app improves anxiety and depressive symptoms. While overall effect sizes were small, at the population level, these can make significant contributions to public mental health.

## Author summary

Increasing burden of anxiety amongst young adults has made widely accessible mobile health applications a promising tool in improving anxiety symptoms at scale. We conducted a randomized controlled trial (N = 323) to examine the effectiveness of a brief, publicly available mobile health application (Intellect’s “Anxiety and Worry” programme) in reducing anxiety and worry symptoms among young adults. Participants who received the intervention showed significant reduction in anxiety and depressive symptoms, however, these effects did not significantly differ from the active control group. At post-intervention, only the Intervention group continued to experience improvements in anxiety level. We also found that higher app engagement with the mHealth app predicted better anxiety and depressive symptom outcomes, and this relationship was fully mediated by psychological mindedness. Future work would benefit from inclusion of a waitlist control group, a larger sample size, and identification of alternative mediators.

## Introduction

Worry is a cognitive-based construct, commonly defined as an uncontrollable chain of thoughts and images about future events that are negatively affect-laden [1,2]. Anxiety, on the other hand, is a multi-encompassing construct defined as an emotion characterized by somatic, cognitive, and behavioral responses [3,4]. Previous research has demonstrated a directional relationship between worry producing anxiety and confirmed the high correlational nature of the two constructs [2,4]. Indeed, worry has been attributed as the cognitive component of anxiety, where persistent and excessive worry is the central diagnostic criteria for generalized anxiety disorder (GAD) [2,5]. Anxiety often co-occurs with depression, a clinical disorder characterized by persistent mood disturbances, negative self-concept, and anhedonia or the inability to experience positive affect [6–8]. Although anxiety and depression have overlapping symptoms, anxiety and depression have distinct temporality (future vs. past oriented) and characteristic (hyperarousal vs. anhedonia) [2,5,6,9]. Anxiety has been associated with an increased risk for cardiovascular illness [9], mortality and suicide [10,11], as well as reduced quality of life [9]

University students are particularly vulnerable to anxiety and depression, as they are exposed to multiple stressors such as major life events (e.g. transition to university life) and societal trends (e.g. increasing financial burden) [12]. A recent meta-analysis of 19 studies involving undergraduates reported a 29% prevalence rate of anxiety and 37% prevalence rate of depression during the pandemic [13]. In addition to its high prevalence rate, anxiety and depressive symptoms are the most common problems for which university students seek help [10,14]. While effective psychological interventions exist for anxiety and depression, only few are utilizing these due to multiple barriers, such as high treatment costs, limited accessibility, lack of perceived need for help, and negative stigma [15–18]. Unfortunately, anxiety and depression usually take a chronic course, and are unlikely to resolve without appropriate interventions [19]. Therefore, there is a dire need for an effective, cost-efficient intervention on anxiety and depression.

One solution to help a large number of people is to utilize widely-accessible, secure yet inexpensive mobile health (mHealth) applications [20–22]. Emerging research has found evidence that engaging with mHealth apps can improve anxiety and depressive symptoms across both clinical [20] and non-clinical populations [23–26]. To date, several meta-analyses have highlighted promising evidence on the success of mHealth apps in decreasing anxiety and depressive symptoms using randomized controlled trials (RCT). As anxiety and depression share overlapping symptoms, some intervention components for anxiety are applicable to depression, such as cognitive restructuring and behavioral activation [27,28]. This is supported by studies of CBT-based mHealth interventions that reported significant improvements on both anxiety and depressive symptoms [29]. Although effect sizes found were small, recent meta-analyses have also highlighted promising evidence on the effectiveness of mHealth interventions on anxiety (g = 0.30–0.38) and depressive symptoms (g = 0.28–0.38) [21,30,31]. However, comparisons against Active Control often yield significantly weaker and inconsistent results [21,30,32]. Nevertheless, scaled at population level, small effects can meaningfully impact public mental health.

Despite promising evidence on the efficacy of mHealth apps on improving anxiety and depressive symptoms, especially for mild symptom severity, there is still a limited number of applications with reported effectiveness [33,34], quality [35], and evidence-based components [36]. Wang et al. [34]’s systematic review found that only 14 out of over 100 studies had reported clinically validated evidence on its effectiveness. In a review of 27 popular apps by Wasil et al. [36], most apps contained just 3 evidence-based components and incorporated limited core treatment techniques typically present in standard anxiety and depression treatment protocols, where cognitive restructuring made up just 12% and 31% of the applications, respectively. Therefore, there is a strong need for more empirical studies to gain a better perspective of the true value of mHealth apps on anxiety as well as depression, paying extra attention to the content used within the application and the type of control groups used in the studies.

While more effort has been made to examine the efficacy of publicly available mHealth apps, limited studies have investigated the underlying mechanisms that enable such impact. To our knowledge, only 5 studies evaluated such mechanisms [23,37–40], and only 3 of these studies utilized mHealth apps incorporating multiple Cognitive Behavior Therapy (CBT) components. Using *MoodMission*, *MoodPrism*, *and MoodKit*, Bakker and colleagues [23,37,38] demonstrated the mediating effect of coping self-efficacy in the relationship between app engagement ratings and anxiety, depressive symptoms, and mental wellbeing outcomes. Bakker and Richard [37] also found a partially mediated effect of emotional self awareness (ESA) in the aforementioned relationship. Identifying additional mechanisms that facilitated improvements in mental health outcomes can further inform mHealth apps developers to create apps that utilize multiple mediators to enhance mental health outcomes [37]. One such potential mediator is psychological mindedness (PM), defined by Denollet and Nyklíček [41] as “the intrinsic motivation to be in touch with one’s inner feelings and thoughts by monitoring and analyzing them in an adaptive way.” An important factor in the development of emotion regulation, PM has previously been linked to lower psychological distress and better coping abilities [42–49]. Previous research has also hinted at the prospect of PM as a relevant skill in psychotherapy, albeit inconsistent findings on its effect on treatment outcomes [44,50–52]. As psychotherapies encourage individuals to monitor, evaluate, and express their cognitions, emotions, and behaviors, it is likely that participating in treatments increases an individual’s PM [53–55]. Nyklíček et al. [54] demonstrated that changes in PM scores over the course of an intervention were associated with larger decreases in psychological symptoms scores, including anxiety, depressive symptoms, and sleeping problems in a clinical sample. Additionally, previous research has indicated that metacognitive skills such as PM are systematically developed during the transition from adolescence to adulthood and are considered key mechanisms required to transform behaviors during cognitive-behavior-based and mindfulness-based treatments [56,57]. Altogether, this suggests that PM might act as a mediator that intermediately affects treatment outcomes from the use of mHealth apps.

### Objectives

This study aimed to evaluate the efficacy of a recently developed mHealth programme within the Intellect mHealth application called (“Anxiety and Worry”) compared to the Active Control group (“Procrastination” programme) among young adults in a randomized controlled trial (RCT) with a 2-week follow-up. We hypothesized that participants in the Intervention group would experience significant decreases in anxiety (primary outcome) and depressive symptoms (secondary outcome) compared to the Active Control group. Based on Bakker and colleagues’ [23,37,38] prior work, we also sought to examine whether PM mediates the hypothesized relationship between engagement with the mHealth programmes and improvements in anxiety and depressive symptoms.

## Materials and methods

### Design

This randomized controlled trial (RCT) is a 2 x 3 mixed factorial experimental design, with condition (Intervention vs. Active Control) as the between-groups factor and time of assessment (pre- intervention vs. post-intervention vs. 2-week follow-up) as the within-groups factor. The study was registered with ClinicalTrials.gov (NCT04911803; see S1 Protocol) and it was approved by the National University of Singapore (NUS)’s Institutional Review Board / NUS-IRB (Reference Code: 2021–266). The study was conducted entirely remotely between June 2021 and December 2021.

### Participants

Participants were recruited through convenience sampling via the NUS’ internal Research Recruitment portal and through the NUS Department of Psychology’s Research Participation (RP) pool. Recruitment took place between June 2021 and December 2021, and potential participants were screened for eligibility via a Qualtrics survey. Inclusion criteria included (1) being able to read and understand English, (2) aged 18 years or above, (3) has not participated in similar mHealth studies. Participants were ineligible if they met any of the following exclusion criteria: (1) 18 years or below, (2) inability to read and understand English, (3) participated in other mHealth studies, (4) lack of mobile device or access to the Apple or Google Play Store, and (5) lack of access to the Internet. Participants who did not complete the baseline questionnaire were also excluded. Only participants who met the inclusion criteria were awarded either $15 reimbursement or 3 course credits for their full participation.

### Materials

Intellect is a publicly available mobile health application aimed at providing mental wellbeing support using self-guided resources. Recently, these self-guided resources have been validated in previous randomized-controlled trials [58,59]. All programmes within the application were developed and have been reviewed by Intellect’s Scientific Advisory Board consisting of clinical psychologists and cognitive behavioral therapists. For the purposes of this study, a version of the Intellect Application is used, where only the “Anxiety and Worry” or “Procrastination” programmes are available, depending on the participant’s assigned condition.

#### “Anxiety and Worry” Programme (Intervention Condition)

**Fig 1:**
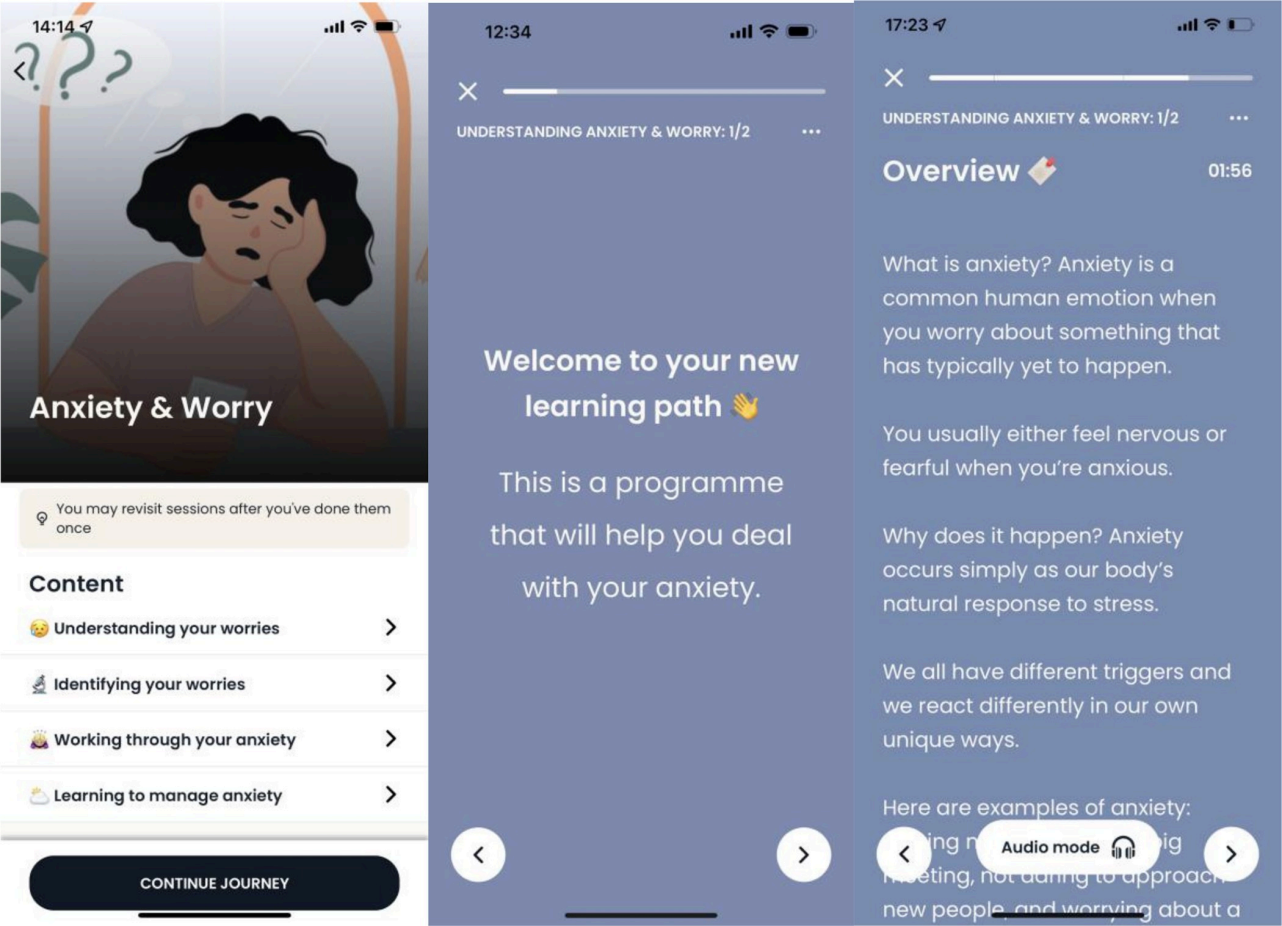
Intellect Anxiety and Worry Programme. The Intellect “Anxiety and Worry” Programme is a free self-guided programme within the Intellect Application.

This is a 14-day mHealth programme within the Intellect Application that provides psychoeducation on the negative core beliefs and/or thought patterns associated with anxiety and worry, as well as effective coping mechanisms and problem-solving techniques (see Fig 1). Each psychoeducation module is followed by short writing exercises, and a few situational-task questions (see Table 1 for a programme overview). The modules and tasks are divided into short sessions, such that participants can engage in a <5 minutes session a day. This module structure allows participants to actively practice utilizing the techniques they have learned.

**Table 1:** Overview of the Anxiety and Worry Programme.

Topics	Content
Understanding your worries	● Understanding Anxiety & Worry ● Learning how to spot Anxiety and what triggers it ● Understanding core beliefs and how it was formed ● Understanding your coping mechanisms
Identifying your worries	● Introduction of the Worry Tree Technique
Working through your Anxiety	● Develop skills to focus on the possible solutions of one’s Anxiety
Learning to manage Anxiety	● Understand what thought patterns are and how to change them ● Recap of the entire programme and key information in each session

#### “Procrastination” Programme (Active Control Condition)

This is a 14-day mHealth programme within the Intellect Application that provides psychoeducation on the procrastination cycle, the mental barriers that arise due to procrastination, and ways to tolerate and overcome the discomfort. The structure and duration of the Procrastination Programme (PP) is similar to that of the Anxiety and Worry programme (see Table 2 for programme overview).

**Table 2:** Overview of the Procrastination Programme.

Topics	Content
Motivations behind Procrastination	● Understanding Procrastination and its cycle ● Learning what mental barriers are, how to spot and how to challenge it
Overcoming the barrier	● Learning and practicing mindfulness ● Identify source of discomfort that leads to Procrastination ● Learning different ways to tolerate discomfort
Creating better habits	● Develop skills to create healthier habits ● Recap of the entire programme and key information in each section

Table 3 describes some examples of the exercises engaged by the participants in each condition.

**Table 3:** Overview of Evidence-Based Treatment Components Covered.

Treatment Component	Example Sub-Components	Anxiety Programme	Procrastination Programme
Psychoeducation	Education on Topic (definition, cycle of reinforcement, etc)	✓	✓
Self-Monitoring	Monitoring Cognition, Emotions, and Behaviors	✓	✓
Cognitive Techniques	Identifying Thoughts, Cognitive Restructuring Exercises	✓	✓
Journaling	Reflective Writing, Informative Journaling	✓	✓*
Behavioral Techniques	Behavioral Activation, Behavioral Experiments	✓*	✓
Problem Solving	Problem Solving Exercise	✓	
Coping Strategies	Identifying coping abilities	✓	
Relaxation Skills	Mindfulness Exercise		✓

*indicates minimal presence in the programme

### Procedures

Participants signed up for the study through the University’s recruitment sites, where they were redirected to a secured Qualtrics survey link. Participants who provided digital consent to participate in the study (N = 492) were prompted to complete the baseline questionnaires, where outcome variables (PHQ-9, GAD-7, PM) were measured. Only participants who were able to download the application, have not participated in similar mHealth app studies, and completed the baseline measures (N = 323) were then automatically randomized using the randomizer function in Qualtrics to the Intervention (“Anxiety and Worry” Programme; N = 160) or the Active Control (“Procrastination” Programme; N = 163) condition. As the study is a single-blind trial, participants were broadly informed that the main purpose of the study was to evaluate the benefits of using a mHealth programme to improve wellbeing [58]. In addition, participants were not informed about the specific details of the study and their assigned condition beforehand in order to lower expectation biases [60]. Next, participants were guided to download the Intellect mHealth application and were provided a unique code to register and access a research version of the app, where only their assigned programme was available. This was done to ensure that any treatment effects observed were due to the programme, and not other features available in the application. Both programmes span over 14 days, with one session to be completed daily (i.e. <5 minutes). After completion of their allocated mHealth programmes, participants were contacted to complete the same outcome variables (PHQ-9, GAD-7, PM) and the app engagement scale (AES). The same measures (but without the AES) were administered 14 days after the completion, and participants were also debriefed about the main purposes of the study via email. A 14 day follow-up was chosen to mimic previous studies [61].

### Measures

#### Primary Outcome Measures

*Generalized Anxiety Disorder Scale (GAD-7)*. Generalized Anxiety Disorder- 7 Scale (GAD-7) is a 7-item self-report instrument that measures anxiety on a continuum [62] and is widely used in research in both clinical and nonclinical populations [63]. Example items include “feeling nervous, anxious, or on edge” and “not being able to stop or control worrying.” Items are scored on a 4-point likert scale, ranging from Not At All (0) to Nearly Every Day (3). Scores of ≥ 10 indicate moderate levels of anxiety [64]. GAD-7 has an excellent internal consistency with Cronbach’s *α* = .84 to *α* = .90 in this study.

#### Secondary Outcome Measures

*Patient Health Questionnaire (PHQ-9)*. Patient Health Questionnaire (PHQ-9) is a 9-item self-report instrument that measures depressive symptomatology. Example items include “little interest or pleasure in doing things” and “feeling tired or having little energy.” Items are scored on a 4-point Likert scale, ranging from Not at all (0) to Nearly Every Day (3), where higher scores indicate more severe depressive symptoms. Scores of ≥ 10 indicate moderate severity of depressive symptoms [65]. PHQ-9 has a good internal consistency with Cronbach’s *α* = .85 to *α* = .86 in this study.

*Psychological Mindedness Scale (PMS)*. Psychological Mindedness Scale (PMS) is a 45-item self-report instrument that measures an individual’s ability to be reflective about interpersonal relationships, psychological processes and meanings across both intellectual and emotional dimensions. Example items include “I often find myself thinking about what made me act in a certain way” and “I am sensitive to the changes in my own feelings.” Items are scored on a 4-point Likert scale ranging from Strongly Agree (4) to Strongly Disagree (1). Consistent with previous research where Cronbach’s *α* = .86 to *α* = .87 [44], the reliability of PMS in this study was acceptable with Cronbach’s *α* = .77 to *α* = .79.

*App Engagement Scale (AES)*. App Engagement Scale (AES) is a 7-item questionnaire developed by Rickard et al. [37,66] based on the Mobile Application Rating Scale’s (MARS) items and themes, such as engagement, functionality, aesthetics, and information [67]. Example items include “overall I was satisfied with the app” and “it had about the right amount of information.” All 7 items are scored on a 5-point Likert scale ranging from Strongly Disagree (1) to Strongly Agree (5). The AES displayed good internal reliability with Cronbach’s *α* = .83 in this study.

### Statistical Analysis

#### Power analysis

Power was calculated with G*Power v.3.1 [68]. Using a small effect size from similar mHealth RCT’s [23,69], setting cronbach alpha at .05, and the power at .90, N = 88 participants per group was required. Accounting for high dropout rates observed in previous studies [23,70], we aimed to recruit a minimum of N = 200 participants.

#### Data cleaning

Data cleaning included examining the data for incompleteness. For missing data, t-tests and chi-square tests were used to compare demographics and outcome measures between complete cases with and without missing data to verify whether or not data were missing completely at random (MCAR). All missing data was then addressed using the last observation carry forward imputation method [71], such that missing data from the post-intervention and follow-up assessment were replaced with data from the last completed questionnaire. The final dataset can be found in the supplementary information of this study (i.e., S1 Data).

All data were analyzed according to the intention-to-treat principle using IBM SPSS v.25. Preliminary data analyses included normality testing and checking for univariate outliers. 13 outliers (i.e. +/- 3SD from the mean) were excluded. Baseline group differences were examined with the independent samples t-tests and chi-square test with a significance level of *p* < .05

#### Main analysis

A 2x3 Mixed Analysis of Variance (Mixed ANOVA) with time (baseline (T1), post-intervention (T2), and follow-up (T3)) as the within subject factor and condition (Anxiety, Procrastination) as the between subject factor was conducted to assess the difference in anxiety (GAD-7) and depressive (PHQ-9) symptom scores. As there were no significant demographic differences (i.e. age, gender) between conditions, no covariates were controlled for in our analysis. *P* levels were corrected for non-sphericity using the Greenhouse-Geisser epsilon when necessary. Significant time by condition interaction effects were further examined using Bonferroni adjusted contrast analyses to compare between group differences on the outcome measures across each time point [72]. A significant interaction effect indicates group differences in improvement over time. Next, a series of mediation analyses were performed using Hayes’ PROCESS macro plugin Model 4 with confidence intervals set at 95% using bootstrapping procedures, with 5000 samples (95% confidence interval) [73]. An effect was considered to be significant if zero was not contained in the confidence interval. Using Zhao et al. [74] and Rucker et al. [75]’s recommendation, mediation was explored even in the absence of a significant total or direct effect. Two mediated regression models were used to examine the relationship between App Engagement Ratings and outcome variables, including Anxiety symptoms (GAD-7) and Depressive symptoms (PHQ-9). The mediating variable used in both models was PM at T2 measured by PMS and the dependent variable used was the outcome variables at T3. No covariates were included in this model. All Beta (β) statistics reported in the regressions are standardized effect sizes.

The main analyses were performed on the whole ITT sample (n = 299), while the mediation analyses were performed on the ITT sample who completed AES (n = 196). Eta squared partial (η^2^_partial_ ≈ .01: small; η^2^_partial_ ≈ .06: medium; η^2^_partial_ ≈ .14: large) was the effect size reported for all findings [76]. The significance level was set as p < .05.

## Results

All results described complied with the CONSORT guidelines for reporting RCTs (see S1 Checklist).

### Baseline Evaluation

No significant baseline group differences on baseline outcome measures, gender, and age, were observed between the intervention and control conditions (p>.05). There were no significant differences in application engagement between both conditions (p>.05), indicating that participants were equally engaged throughout their respective programmes. The high mean scores on application engagement seen across both conditions (Intervention: M = 27.29, SD = 3.81; Active Control: M = 27.04, SD = 3.66) may infer that participants generally found our application to be acceptable [38,58]. At baseline, the sample was mildly anxious (M = 7.25, SD = 4.95) and depressed (M = 7.51, SD = 5.33), where 92 participants (30.77%) had a GAD-7 score of 10 or over, indicating moderate levels of anxiety and 90 participants (30.10%) had a PHQ-9 score of 10 or over, indicating moderate severity of depressive symptoms. There were no significant baseline group differences observed for the number of participants that experienced moderate levels of anxiety and depressive symptoms. There were also no significant differences in application engagement between these participants in both conditions [p>.05]. Sample characteristics for the final sample of 299 participants (M_age_ = 22.05 years; SD_age_ = 4.06 years, range: 18 to 54 years) are displayed in Table 4.

**Table 4:** Baseline Characteristics.

Variables		Intervention Condition (N = 150)	Active Control Condition (N = 149)	*p-value*
		*M (SD)*	*M (SD)*	
**Age in years**		22.160 (4.324)	21.940 (3.877)	0.643
**GAD-7**		7.553 (4.894)	6.946 (5.005)	0.453
**PHQ-9**		7.573 (5.347)	7.436 (5.928)	0.824
**PMS**		126.260 (10.063)	127.215 (9.982)	0.586
AES**		27.286 (3.813)	27.041 (3.661)	0.878
		***N (%)***	***N (%)***	
**Gender**	**Female**	118 (78.67%)	105 (70.47%)	0.259
	**Male**	31 (20.67%)	43 (28.68%)	
	**Others**	1 (0.67%)	1 (0.67%)	
**Participants with GAD-7/PHQ-9 score** ≥ 10*	**GAD-7**	52 (34.67%)	40 (26.85%)	0.143
	**PHQ-9**	45 (30.00%)	45 (30.20%)	
	**Comorbid**	35 (23.33%)	30 (20.13%)	

GAD-7 = Generalized Anxiety Disorder; PHQ-9 = Patient-Health Questionnaire; PMS = Psychological Mindedness Scale; AES** = Application Engagement Scale, collected at post-intervention

*A GAD-7/PHQ-9 score of 10 or more indicates moderate levels of anxiety/depressive symptoms

### Dropout Analysis

**Fig 2:**
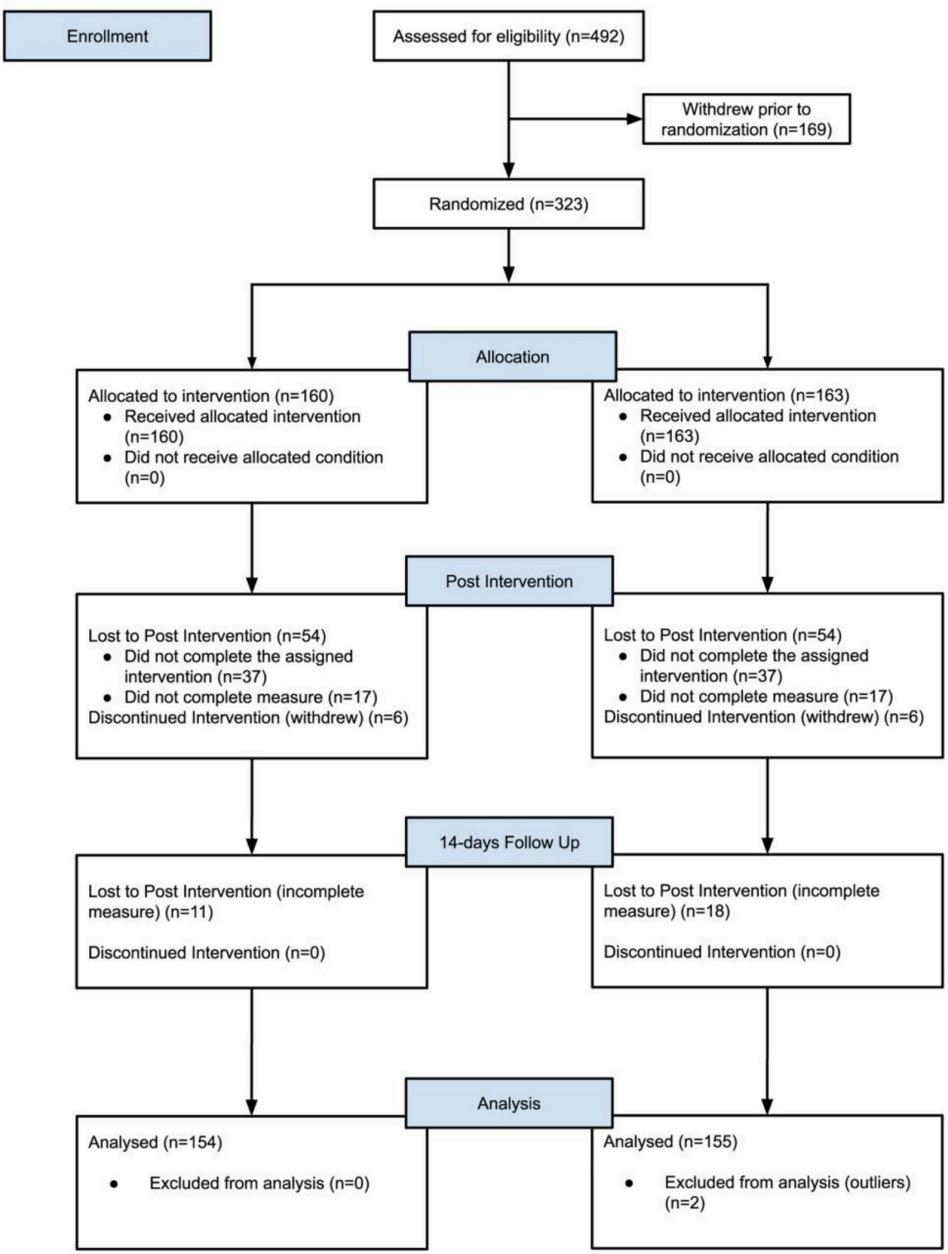
CONSORT Flow Diagram.

In total, 323 participants were randomized to either the Intervention or Active Control condition (See Fig 2). Of these, 11 participants withdrew prior to the post-intervention assessment. Both groups also experienced attrition during the two-week intervention period (35.48% of the Intervention condition and 34.49% of the Active Control condition). Additionally, 7.74% of the Intervention condition and 12.10% of the Active Control condition were lost to follow-up. Participants who dropped out during the study period did not significantly differ in age, gender, anxiety symptoms, depressive symptoms, application engagement and psychological mindedness scores compared to participants who did not drop out (all ps>.05).

Adherence to the study was defined as completing the assigned programme (when applicable) and questionnaires. By this definition, 65.06% (203/312) adhered to the study at T2 and 55.12% (172/312) adhered to the study at every time point. There are no significant differences in adherence at each timepoint between groups (p>.05).

### Test of Group Effects

The mean score and standard deviation for each outcome measure by condition and time point are shown in Table 5. Mixed ANOVA with anxiety (W(2) = .890, p < .001) and depressive symptoms (W(2) = .794, p < .001) indicated that the assumption of sphericity had been violated, therefore the Greenhouse-Geisser corrected values were reported. Significant main effects of time were found for both outcome measures, where both conditions improved in anxiety and depressive symptoms over time (Anxiety symptoms: F(1.802,535.19) = 12.306, p < .001, ηp^2^ = .040; Depressive symptoms: F(1.658,492.365) = 16.984, p < .001, ηp^2^ = .054). Results from subsequent pairwise comparisons revealed a significant mean difference from baseline (Anxiety symptoms: M = 7.553, SD = .404; Depressive symptoms: M = 7.573, SD = .436) to post-intervention (Anxiety symptoms: M = 7.140, SD = .345, p < .001, 95% C.I. = [.232, 1.152]; Depressive symptoms: M = 6.807, SD = .408, p < .001, 95% C.I. = [.354, 1.291]) and from baseline to follow-up (Anxiety symptoms: M = 6.613, SD = .357, p < .001, 95% C.I. = [.401, 1.453]; Depressive symptoms: M = 6.453, SD = .409, p < .001, 95% C.I. = [.523, 1.563]), where the Active Control condition reported lower mean on anxiety and depressive symptoms in each instance.

**Table 5:** Comparison of Group Effects for Anxiety symptoms (GAD-7) and Depressive symptoms (PHQ-9) across timepoints.

	Intervention			Active Control			Baseline (T1) to Follow-Up (T3)		
**Outcome Variable**	**T1 (M, SD)**	**T2 [after 2 weeks] (M, SD)**	**T3 [after 4 weeks] (M, SD)**	**T1 (M, SD)**	**T2 [after 2 weeks] (M, SD)**	**T3 [after 4 weeks] (M, SD)**	**Time**	**Condition**	**Time x Condition**
**GAD-7**	7.553 (4.894)	7.140 (4.161)	6.613 (4.319)	6.946 (5.005)	5.973 (4.296)	6.242 (4.425)	**F(1.802,535.194) = 12.306, p<0.001, ηp**^**2**^ **= 0.040**	F(1,297) = 2.206, p = 0.139, ηp^2^ = 0.007	F(1.802,535.194) = 2.624, p = 0.079, ηp^2^ = 0.009
**PHQ-9**	7.573 (5.347)	6.807 (4.901)	6.453 (4.776)	7.436 (5.330)	6.349 (5.092)	6.470(5.201)	**F(1.658,492.365) = 16.984, p < .001, ηp**^**2**^ **= 0.054**	F(1,297) = 0.100, p = 0.724, ηp^2^ = 0.000	F(1.658,492.365) = 0.760, p = 0.445, ηp^2^ = 0.003

Note: Bolded text indicates a significant p-value at .05.

GAD-7 = Generalized Anxiety Disorder

PHQ-9 = Patient Health Questionnaire

*p < .05

**p < .01

***p < .001Mediation Analyses

**Fig 3:**
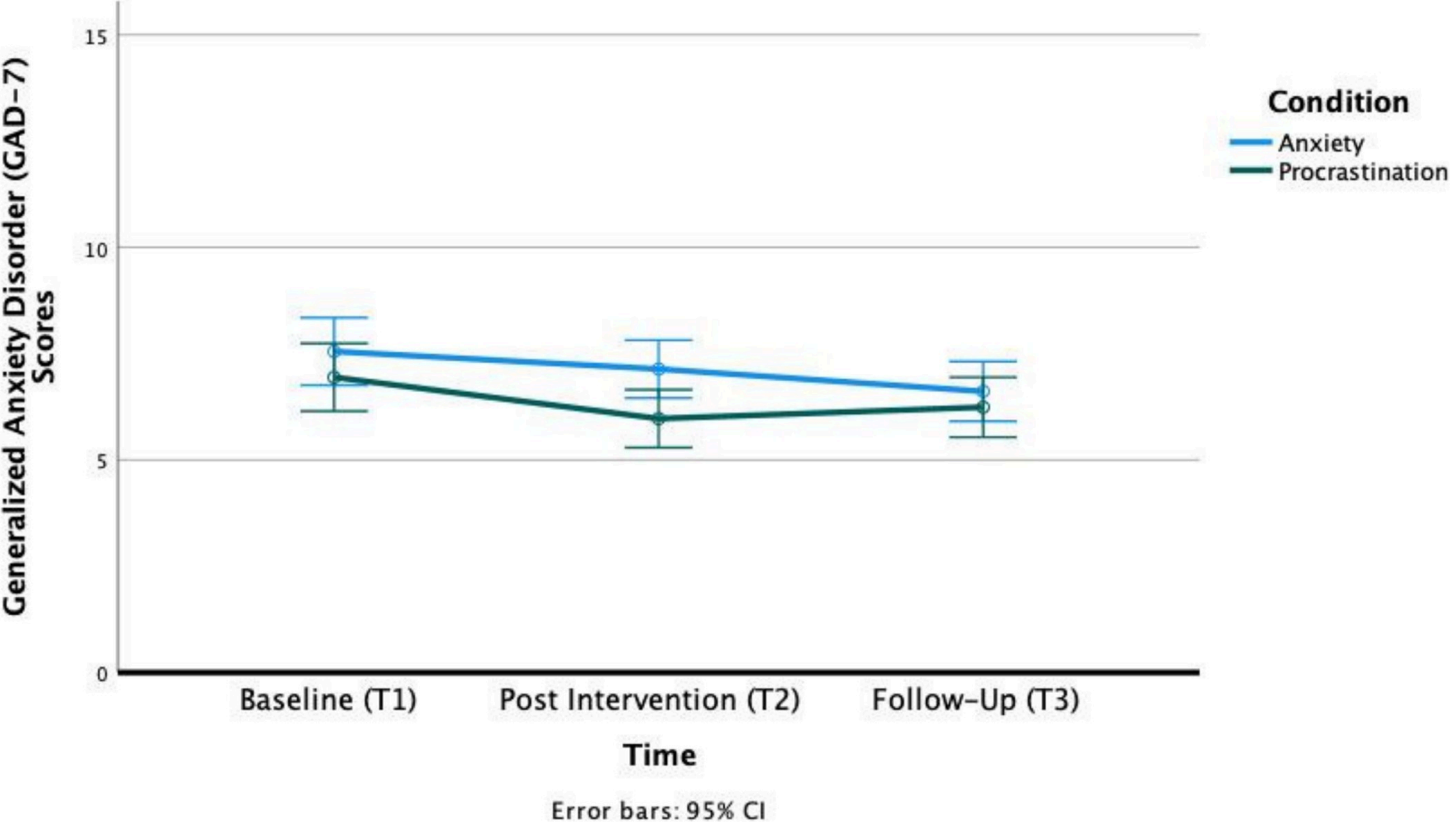
Profile Plot for changes on the GAD-7 for both groups across timepoints. Error bars represent 95% CIs.

While there were no significant effects of time by condition for anxiety and depressive symptoms (Anxiety symptoms: F(1.802,535.194) = 2.624, *p* = .079, ηp^2^ = .009; Depressive symptoms: F(1.658,492.365) = .760, p = .445, ηp^2^ = .003), results from post-hoc pairwise comparisons revealed a significant decrease in anxiety symptoms from post-intervention to follow-up for the Intervention condition (*p* = .033, 95% C.I. = [.031, 1.023]) and a non-significant increase in anxiety symptoms for the Active Control condition (p = .585, 95% C.I. = [-.766, .229]). Although both conditions experienced a significant decrease in anxiety symptoms from baseline to follow-up (Intervention: p = .002, 95% C.I. = [.279, 1.601]; Active Control: *p* = .033, 95% C.I. = [.041, 1.368]), only the Active Control condition reported a significant decrease from baseline to post-intervention (p = .001, 95% C.I. = [.324, 1.622]). Altogether, this indicated that the Intervention condition experienced significant improvements at follow-up driven by continued improvements from post-intervention to follow-up, while the Active Control condition experienced significant improvement at post-intervention with no significant improvements from post-intervention to follow-up (See Fig 3). For depressive symptoms, post-hoc pairwise comparisons revealed a significant decrease from baseline to post-intervention (Intervention: p = .041, 95% C.I. = [.023, 1.510]; Active Control: p = .002, 95% C.I. = [.342, 1.3833]) and from baseline to follow-up (Intervention: p = .005, 95% C.I. = [.230, 1.703]; Active Control: p = .033, 95% C.I. = [.041, 1.368]) for both conditions. This indicated that both conditions did not significantly differ in the improvement trajectory of depressive symptoms.

There were no significant main effects of condition observed for both outcome measures (p>.05). The full results of the analysis are presented in Table 5.

**Fig 4:**
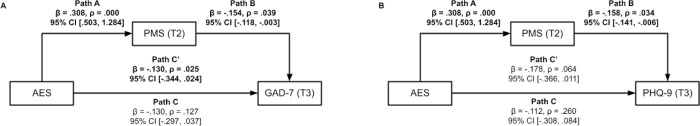
**Mediated regression models using AES as the predictor and (A) Anxiety symptoms and (B) Depressive symptoms as the outcome.** Whole sample (N = 196) mediated regression models using App Engagement ratings (AES) as the predictor and (A) Anxiety symptoms (GAD-7), (B) Depressive symptoms (PHQ-9) scores as the outcome, where T2 indicates post-intervention and T3 indicates 2-week follow-up. Note: bolded coefficients indicate significance, p < .05.

Fig 4 shows the full results of the 2 mediation analyses performed. A significant total effect between AES and Anxiety scores was observed, β = -.184, t_196_ = -2.268, *p* = .025. However, there were no significant total effects of AES on Depressive symptoms β = -.178, t_196_ = -1.863, *p* = .064. No direct effects were significant. Although results revealed an absence of significant total effects for Depressive symptoms, mediation analyses were carried out for both outcome measures as per Zhao et al. [73]. Mediation analyses revealed significant standardized indirect effects of AES through PMS for Anxiety symptoms, β = -.047, 95% CI [-.106, -.007] (See Fig 4A), and Depressive symptoms, β = -.049, 95% CI [-.105, -.007] (See Fig 4B). Overall, these models accounted for 4.72% and 1.76% of the variance (*R*^*2*^) in anxiety and depressive symptoms reduction, respectively. Results indicated the presence of an indirect-only mediation of PMS at T2 for both anxiety and depressive symptoms at T3. This suggests that AES causes a decrease in anxiety and depressive symptoms indirectly by increasing PMS.

## Discussion

This study sought to evaluate the efficacy of Intellect’s mHealth programme “Anxiety and Worry” in improving anxiety and depressive symptoms in college students. The tools implemented in the intervention are based on cognitive-behavior therapy principles, and include cognitive, behavioral, and writing activities that may increase an individual’s ability to self-reflect and introspect. Thus, we also examined PM as mediators of app engagement on anxiety and depressive symptoms.

### Efficacy of the Anxiety and Worry Programme

Our first hypothesis was not fully supported. Even though the “Anxiety and Worry” programme is based on CBT principles used in anxiety interventions, participants in the “Anxiety and Worry” programme did not experience significantly greater improvements in anxiety and depressive symptoms compared to participants in the “Procrastination” programme. Rather, participants in both conditions displayed a significant decrease in levels of anxiety and depressive symptoms from baseline to follow-up. While there was no significant Time by Condition interaction on depressive symptoms, the present study showed a marginally significant Time by Condition interaction on anxiety scores, with participants in the Active Control condition reporting lower mean scores at follow-up. Further examinations demonstrated that anxiety symptoms significantly decreased after the completion of the “Anxiety and Worry” programme, i.e. from post-intervention to follow-up, whereas no such continued improvement was observed in the Active Control condition. The result from the analysis is in line with other follow-up studies that reported continued improvement of anxiety symptoms among individuals in the intervention group after the intervention ceased [77,78]. One possible explanation for this observation is Rachman’s (2017) notion that the full benefits of CBT-based intervention on anxiety will only manifest post-intervention, after participants fully understood the skills taught and practiced applying them in their day-to-day lives [79].

Altogether, the results did not demonstrate the superiority of the “Anxiety and Worry” programme to the “Procrastination” programme. However, when compared with previous studies that evaluate the efficacy of mHealth interventions against Active Control on anxiety and depressive symptoms among the general population, non-significant results were mostly reported [61,80]. SuperBetter (SB), a game-like mobile-based self-help programme, required participants aged 18 years or older to engage with the application at least 10 minutes per day for a month [61]. Roepke et al. [61] found that the intervention condition (SB with CBT and positive psychotherapy content) was more effective in reducing depressive and anxiety symptoms at post-intervention and follow-up, but not the Active Control condition (i.e. General SB application). Since this study was conducted during the COVID-19 pandemic (between June and December 2021) which may explain the increased anxiety and depressive symptoms at baseline [81–83], it is also possible that the higher baseline symptoms hindered the efficacy of the “Anxiety and Worry” intervention. For instance, McKee et al. (2007) found that individuals with a higher tendency to experience negative affectivity symptoms (i.e., anxiety and depression) were less able to engage meaningfully with the intervention and suffered from less than optimal outcomes [84]. Nevertheless, even though the “Anxiety and Worry” programme was not superior to the “Procrastination” programme in terms of lowering anxiety and depressive symptoms, it is encouraging that participants in both conditions reported a significant decline in anxiety and depressive symptoms at follow-up. While we cannot rule out the effect of third variables, it is plausible that both mHealth programmes are effective in reducing anxiety and depressive symptoms.

One explanation for the unexpectedly good outcomes in the active control group is that the procrastination mHealth programme is also based on the cognitive-behavior model and thus contains multiple techniques that may be beneficial for anxiety and depressive symptoms. For example, one module in the programme is titled “Overcoming the barrier” and contains discomfort intolerance (DI) related information and relaxation exercises targeting participants’ abilities to overcome difficult scenarios. The module aims to equip participants with the necessary skills to overcome similar scenarios in the future by teaching them to be mindful and walking them through how to challenge procrastination. Previous studies have recognized DI as central to anxiety pathology [85] In particular, discomfort avoidance, a component of DI, is primarily responsible for anxious responding [85,86]. Therefore, it is likely that this module helped participants to improve DI and thus decreased anxiety. Moreover, this module incorporates exposure tasks related to procrastination. Peris et al. [87] previously highlighted that exposure tasks in CBT treatment were often followed by significant improvement in the rate of progress in treatment, including the primary treatment outcome (i.e. procrastination) and secondary treatment outcome (i.e. anxiety). Additionally, the exposure tasks included in this module include reducing avoidance, such as encouraging participants to complete the task they previously put off. Such tasks involve principles of behavioral activation (BA), an established technique recognized as important in depression interventions [88,89]. Taken together, this suggests practical implications for future developments of mHealth programmes, in terms of including modules that address discomfort intolerance related to anxious responding and incorporating elements of exposure tasks and behavioral activation that likely help improve anxiety and depressive symptoms.

Furthermore, helping students with procrastination may have inadvertently improved anxiety and depressive symptoms more than we had anticipated. Previous studies have indicated that 50–70% of college students showed frequent procrastination behavior [90] and linked procrastination to negative well-being outcomes, including higher anxiety and depressive symptoms [91]. Indeed, a mediating effect of rumination between anxiety and procrastination as well as depression and procrastination was previously observed among students [92]. Constantin et al. [92] indicated that students experiencing anxiety and depressive symptoms might more readily procrastinate in order to manage their current distress or regulate their negative mood states. Thus, it is highly plausible that interventions targeting procrastination may improve one’s ability to manage psychological distress, especially in the student population. This is supported by previous meta-analysis that found a small effect size of procrastination intervention on anxiety and depressive symptoms among a mix of students and the general population [93]. Therefore, using PP as an Active Control may have exerted unexpected benefits on anxiety and depressive symptoms.

Nevertheless, it is encouraging that participants in the intervention condition continued to experience improvements in anxiety from post-intervention (T2) to follow-up (T3), while no such benefit was observed in the Active Control condition. This suggests a potential delayed effect of the “Anxiety and Worry” programme, where full treatment benefits were only manifested post-intervention. One explanation for the apparent delayed effect observed is the major inclusion of writing activities (>80% of activities) within the Anxiety programme. The writing activities are framed as an interactive journal (IJ) using language that elicits change strategies, thereby mimicking motivational interviewing (MI) in writing [94]. The use of MI enhances a “client-as-expert” stance, which indirectly confers a sense of autonomy and induces a belief in clients to rely on their ability to address the situation they are in. Existing MI literature has attributed this mechanism as the source of delay or the sleeper effect at post-intervention [95,96]. A relevant study by Westra et al. [97] found a steeper rate of worry decline at post-intervention for MI-CBT groups. Thus, using the same principles, it is possible that the MI-based IJ components in the Anxiety programme instills a “user-as-expert” stance, which reinforce the participants’ confidence in applying the techniques learnt to address anxiety and worry, ultimately improving their anxiety symptoms post-intervention. This suggests practical implications for future developments of anxiety related mHealth application, in terms of integrating MI-based journaling elements which could aid in long-term effective reductions of anxiety.

### Psychological Mindedness as a Mediator

To our knowledge, this is the first study that investigated Psychological Mindedness as a mediator in the context of mHealth apps. Our second hypothesis is fully supported. The results of our mediation analyses demonstrated that while app engagement did not directly predict reductions in anxiety and depressive symptoms, a mediated pathway was identified via PMS. While effect sizes are small, results of the current study suggest that engaging with the “Anxiety and Worry” and “Procrastination” programmes improved one’s PM, which subsequently decreased one’s anxiety and depressive symptoms. These findings extend previous studies by Bakker’s group [23,37,38] that have observed the mediating role of coping self-efficacy (CSE) on anxiety and depressive symptoms. Previous studies have hinted upon the potential relationship between PM and CSE, particularly problem-focused or adaptive coping [54]. Pang et al. [46] have also established that the insight component of PM significantly mediates the relationship between dysfunctional coping and depressive symptoms. Hence, it is unsurprising to find that PM plays a mediating role in the relationship between App Engagement and anxiety and depressive symptoms.

The present study contributes to the growing literature of PM and CBT-based treatment. Previously, Kishon et. al. [98] established an association between PM and depressive symptoms across timepoints and demonstrated that individuals who are psychologically minded but do not have access to their feelings benefited from CBT treatment. Kishon et al. [98] also suggested the notion that techniques used in CBT-based treatment (e.g. monitoring thoughts, feelings, and behaviors) likely targets the same cognitive analytical abilities that underlie PM and that PM likely assists individuals on the process of emotion regulation during treatments on anxiety and depression. Besides that, previous studies have found PM to be associated with higher commitment and stronger involvement in therapy [37,89,90]. Thus, it is possible that an increase in PM influenced participants’ commitment and motivation to therapy, and the sustained motivation contributes to the lasting improvements in anxiety and depressive symptoms at follow-up.

However, the absence of direct effects of AES on Anxiety and Depressive symptoms might indicate the presence of an unmeasured conflicting mediator that ultimately cancels out the overall direct effect [74,99]. This is highly probable, as the current study did not measure potential negative side effects of the trial such as rumination, which is positively associated with anxiety and depressive symptoms [23,37,38]. Further research is needed to investigate other possible mediators which may have induced an undesired impact on Anxiety and Depressive symptoms to prevent unwanted effects of mHealth programmes.

Nevertheless, the present finding supported the aforementioned theory on the potential reason behind the observed delayed effect in Anxiety as well as the importance of discomfort tolerance in anxiety pathology. A study by Beitel et al. [43] found associations between high PM individuals and their ability to tolerate distressing elements of psychotherapy. Additionally, Beitel et al. [100] also found that highly psychologically minded individuals appear to expect more self-involvement in the treatment process and outcome. Hence, speculatively, it may be that individuals who engaged with the application experienced improvements in PM, which in turn allowed them to better tolerate discomfort and become intrinsically motivated to engage with the app’s “user-as-expert”-inducing features.

### Limitations and Future Directions

Our study is not without limitations. First, no waitlist condition was included. Although our main objective was to assess whether the “Anxiety and Worry” programme is more effective than the active control (“Procrastination” programme) in reducing anxiety and worry, including a passive control would have yielded a clearer result pattern. Secondly, it is possible that the improvements in anxiety and depressive symptoms observed across conditions were caused by performance expectancy, or an individual’s beliefs about the benefit of using information and communications technology, rather than the use of the Intellect mHealth programmes. Previous studies have also found evidence that performance expectancy was positively associated with intention to use mHealth apps [101]. Hence, while an Active Control condition was used to limit the presence of digital placebo, as per Gruszka et al. [102]’s recommendation, simply participating in the study and engaging in mental wellbeing topics might contribute to the improvement of anxiety and depressive symptoms [103]. Third, we underestimated the relevance of CBT techniques utilized in the “Procrastination” programme for anxiety outcomes and thus did not expect that it would be as effective in reducing anxiety among young adults. For future comparison, researchers may consider utilizing active control designs that do not involve CBT-based techniques, such as attention control designs or designs based on different treatment modalities.

Fourth, the present study lacks objective measures of engagement and adherence. While no significant differences were found on the subjective AES measure, the present study did not collect the duration spent on the Intellect Application. Moreover, study adherence was heavily reliant upon completions of the self-reported outcome measures. Even though programme completion was verified with the Intellect database and participants who did not complete the assigned programme were identified, the lack of data on duration spent on the application limits the accuracy of adherence measurement. Therefore, future studies may benefit from including a more objective app engagement measure to limit its potential in acting as a third variable when examining mHealth application’s efficacy. Additionally, approximately 30% of the sample population met the cutoff (≥10) for moderate anxiety and depressive symptoms at baseline. Thus, the present results may not readily be generalized to the clinical population of individuals experiencing anxiety or depressive disorders. Lastly, although the present study provides valuable insight into potential pathways of improvements in anxiety and depressive symptoms via psychological mindedness when engaging in mHealth applications, future research should aim to identify additional mediators that might strengthen the benefit of mHealth applications.

## Conclusion

The intervention and active control showed significant improvements in anxiety and depressive symptoms across time, however both groups did not differ significantly from one another. Nonetheless, our study demonstrated the continual improvement of anxiety induced by the Anxiety programme post-intervention. Altogether, this study suggests that engagement with a CBT-based mHealth programme is related to reduction in anxiety and depressive symptoms. One mechanism involved in exerting this effect is the app’s ability to improve an individual’s Psychological Mindedness. Higher engagement rating of the mHealth programme predicted improvements on anxiety and depressive symptoms. Despite small effect sizes observed, the practical implications of these cost-effective, highly accessible mHealth programmes can meaningfully impact public mental health at scale.

## Supporting information

S1 Checklist CONSORT Checklist. (PDF)

S1 Protocol ClinicalTrial.gov Protocol. (PDF)

S1 Data Final Dataset. (XLSX)
