# Supplementary material for: Efficacy of Intellect’s self-guided anxiety and worry mobile health programme: A randomized controlled trial with an active control and a 2-week follow-up
Source: PLOS Digit Health. 2023 May 24;2(5):e0000095. doi: 10.1371/journal.pdig.0000095 (PMC10208477; doi:10.1371/journal.pdig.0000095)
Supplement: S1 Protocol — (PDF) [file pdig.0000095.s002.pdf]

Try the modernized ClinicalTrials.gov beta website. Learn more about the modernization effort.

U.S. National Library of Medicine
ClinicalTrials.gov

Self-help App and Wellbeing

The safety and scientific validity of this study is the responsibility of the study sponsor and investigators. Listing a study does not mean it has been evaluated by the U.S. Federal Government. Read our disclaimer for details.

ClinicalTrials.gov Identifier: NCT04911803

Recruitment Status : Completed
First Posted : June 3, 2021
Last Update Posted : July 26, 2022

Sponsor:
VTan

Collaborator:
The Intellect Company

Information provided by (Responsible Party):
VTan, National University, Singapore

Study Details | Tabular View | No Results Posted | Disclaimer | How to Read a Study Record

Study Description

Go to

Brief Summary:

Anxiety and worry are amongst the most common mental health difficulties. The Second Mental Health Study found a significant increase in the lifetime prevalence for GAD, from 0.9% to 1.6%. In addition, the Organisation for Economic Cooperation and Development found that among Singaporean students, 86% experienced anxiety levels that were significantly higher than the OECD average.

Past research revealed that individuals who worried more experienced decreases in life satisfaction. It was also found that worry and anxiety are significant predictors of one's psychological wellbeing .

In line with this trend, the market for wellbeing apps have been one of the fastest growing categories of apps ever since; with more than 10,000 on the market. Studies have shown that the use of wellbeing apps has been correlated with an improvement in mental wellbeing. However, due to the lack of research that focuses on disorder-specific evidence, there still exists debates around the effectiveness of wellbeing apps on anxiety and worry. In addition, the lack of research on the mediating factor of psychological mindedness in the relationship between the use of wellbeing apps and mental well-being, could be pivotal to the effectiveness of wellbeing apps.

To show the effectiveness of evidence-based wellbeing apps in targeting anxiety and worry, this study will employ the engagement of participants with a wellbeing app for a controlled period of 2 weeks before recording their mental wellbeing outcomes.This paradigm has been replicated extensively through multiple studies.

This study will use a between-groups experimental study design whereby participants will be block randomised into 2 conditions: Active control condition, and Anxiety condition. Each condition will be given a restricted version of the wellbeing app according to their treatment groups. Follow-up data will be collected at 2-weeks post intervention to establish efficacy of the intervention.

Objective 1: To evaluate the effectiveness of a wellbeing app self-help programme for reducing anxiety and worry.

Objective 2: To examine if psychological mindedness moderates hypothesised effects of wellbeing app usage and anxiety and worry.

Hypothesis 1: Participants in the intervention group will report significantly lower anxiety and worry than participants in the control group.

Hypothesis 2: Psychological mindedness will moderate the effect of the wellbeing app's self-help programme on anxiety and worry: Participants high in psychological mindedness will benefit more from the wellbeing apps than those with lower scores on psychological mindedness.

Condition or disease	Intervention/treatment	Phase
Anxiety and Worry	Behavioral: Anxiety Application<br>Behavioral: Procrastination Application	Not Applicable

Study Design

Go to

Study Type :
Interventional (Clinical Trial)

Actual Enrollment :
492 participants

Allocation:
Randomized

Intervention Model:
Parallel Assignment

Masking:
Triple (Participant, Care Provider, Outcomes Assessor)

Primary Purpose:
Supportive Care

Official Title:
Does Using a Self-help App to Improve Wellbeing Work? - An Experimental Follow-up Study

Actual Study Start Date :
June 8, 2021

Actual Primary Completion Date :
February 8, 2022

Actual Study Completion Date :
April 8, 2022

Resource links provided by the National Library of Medicine

NIH NLM

MedlinePlus related topics: Anxiety

U.S. FDA Resources

Arms and Interventions

Go to

Arm	Intervention/treatment
Experimental: Anxiety Group<br>Participants will download the anxiety application to their own handphones, and will complete the programme subsequently in their own time over the course of 2 weeks. The expected duration participants will spend on the anxiety application daily is estimated to be around 5 to 10 minutes, amounting to a total of around 140 minutes (2 hours and 10 minutes) during the 2 weeks intervention. Participants in the anxiety group are asked to complete daily brief exercises. For example, they will practice noticing worry thoughts and journal them down.	Behavioral: Anxiety Application<br>The Intellect mobile app (containing the anxiety application) can be freely downloaded from the App store and Google Play store for free. The app provides access to the different programmes. The mobile app has been pilot tested by Intellect before the release on the App and Google Play store. However, for this research project Intellect is going to provide the the programmes as stand alone applications exclusively for the participants of this research.

Arm	Intervention/treatment
Active Comparator: Procrastination Group<br>Participants will download the procrastination application to their own handphones, and will complete the programme subsequently in their own time over the course of 2 weeks. The expected duration participants will spend on the procrastination application daily is estimated to be around 5 to 10 minutes, amounting to a total of around 140 minutes (2 hours and 10 minutes) during the 2 weeks intervention. Participants in the procrastination group are asked to complete daily brief exercises. For example, they will practice to reduce procrastination-related thoughts.	Behavioral: Procrastination Application<br>The Intellect mobile app (containing the procrastination application) can be freely downloaded from the App store and Google Play store for free. The app provides access to the different programmes. The mobile app has been pilot tested by Intellect before the release on the App and Google Play store. However, for this research project Intellect is going to provide the the programmes as stand alone applications exclusively for the participants of this research.

Outcome Measures

Go to

Primary Outcome Measures :

1. General Anxiety Disorder - 7 (GAD-7) [Time Frame: Measured before the intervention]

   General Anxiety Disorder- 7 (GAD-7) is a 7 item self report instrument that measures anxiety and is widely used in research in clinical and nonclinical practices anxiety as a continuum (Siddaway, Taylor & Wood, 2018) and is a reliable and valid instrument for assessing anxiety in a general population (Löwe et al., 2008). Items are scored on a 4-point scale, ranging from "not at all (0)", "several days (1)", "more than half the days (2)" and "nearly everyday (3)". GAD-7 produced an excellent internal consistency of α=0.92 in a sample of 2982 participants (Spitzer, Kroenke, Williams, & Löwe, 2006).

2. General Anxiety Disorder - 7 (GAD-7) [Time Frame: 1-2 days after completing the intervention (2 weeks)]

   General Anxiety Disorder- 7 (GAD-7) is a 7 item self report instrument that measures anxiety and is widely used in research in clinical and nonclinical practices anxiety as a continuum (Siddaway, Taylor & Wood, 2018) and is a reliable and valid instrument for assessing anxiety in a general population (Löwe et al., 2008). Items are scored on a 4-point scale, ranging from "not at all (0)", "several days (1)", "more than half the days (2)" and "nearly everyday (3)". GAD-7 produced an excellent internal consistency of α=0.92 in a sample of 2982 participants (Spitzer, Kroenke, Williams, & Löwe, 2006).

3. General Anxiety Disorder - 7 (GAD-7) [Time Frame: 2 weeks after the completion of the intervention]

General Anxiety Disorder- 7 (GAD-7) is a 7 item self report instrument that measures anxiety and is widely used in research in clinical and nonclinical practices anxiety as a continuum (Siddaway, Taylor & Wood, 2018) and is a reliable and valid instrument for assessing anxiety in a general population (Löwe et al., 2008). Items are scored on a 4-point scale, ranging from "not at all (0)", "several days (1)", "more than half the days (2)" and "nearly everyday (3)". GAD-7 produced an excellent internal consistency of α=0.92 in a sample of 2982 participants (Spitzer, Kroenke, Williams, & Löwe, 2006).

4. Patient Health Questionnaire - 9 (PHQ-9) [Time Frame: Measured before the intervention]

   Patient Health Questionnaire (PHQ-9) i s a 9 item self report instrument that measures depression. Items are scored on a 4-point scale, ranging from "not at all (0)", "several days (1)", "more than half the days (2)" and "nearly everyday (3)". In a sample of 3000 patients in the PHQ Primary Care Study, PHQ-9 achieved an excellent internal consistency of α=0.89 (Kroenke, Spitzer, & Williams, 2001).

5. Patient Health Questionnaire - 9 (PHQ-9) [Time Frame: 1-2 days after completing the intervention (2 weeks)]

   Patient Health Questionnaire (PHQ-9) i s a 9 item self report instrument that measures depression. Items are scored on a 4-point scale, ranging from "not at all (0)", "several days (1)", "more than half the days (2)" and "nearly everyday (3)". In a sample of 3000 patients in the PHQ Primary Care Study, PHQ-9 achieved an excellent internal consistency of α=0.89 (Kroenke, Spitzer, & Williams, 2001).

6. Patient Health Questionnaire - 9 (PHQ-9) [Time Frame: 2 weeks after the completion of the intervention]

   Patient Health Questionnaire (PHQ-9) i s a 9 item self report instrument that measures depression. Items are scored on a 4-point scale, ranging from "not at all (0)", "several days (1)", "more than half the days (2)" and "nearly everyday (3)". In a sample of 3000 patients in the PHQ Primary Care Study, PHQ-9 achieved an excellent internal consistency of α=0.89 (Kroenke, Spitzer, & Williams, 2001).

Secondary Outcome Measures :

1. Psychological Mindedness Scale [Time Frame: Measured before the intervention]

   Psychological Mindedness Scale (PM): 45 item self report instrument that measures an individual's ability to be reflective about interpersonal relationships, psychological processes and meanings across both intellectual and emotional dimensions. Items are scored on a 4 point-scale ranging from "strongly agree (4)" to "strongly disagree (1)". The PM has a good internal consistency of α= 0.86 and recomputed to 0.87 in a study of 256 patients (Conte et al., 1990).

2. App Engagement Scale [Time Frame: Measured 1-2 days after completing the intervention (2 weeks)]

   App Engagement Scale (AES): 7 item self report feedback instrument that indicates the degree to which an individual engages with an app. Items are scored on a 5 point-scale ranging from "strongly agree (5)" to "strongly disagree (1)". This App Engagement Scale had good internal reliability, Cronbach's α = .839 in a study of 1349 app user (Rickard & Bakker, 2019).

Eligibility Criteria

Go to

Information from the National Library of Medicine

NIH NLM

Choosing to participate in a study is an important personal decision. Talk with your doctor and family members or friends about deciding to join a study. To learn more about this study, you or your doctor may contact the study research staff using the contacts provided below. For general information, [Learn About Clinical Studies.](#)

Ages Eligible for Study:
18 Years and older (Adult, Older Adult)

Sexes Eligible for Study:
All

Accepts Healthy Volunteers:
No

Criteria

Inclusion Criteria:

- At least 18 years old for NUS students, or 21 for non NUS students

Exclusion Criteria:

- Participants who do not meet the age requirement

Contacts and Locations

Go to

Information from the National Library of Medicine

NIH NLM

To learn more about this study, you or your doctor may contact the study research staff using the contact information provided by the sponsor.

Please refer to this study by its ClinicalTrials.gov identifier (NCT number): ***NCT04911803***

Locations

Singapore

National University of Singapore
Singapore, Singapore, 119077

Sponsors and Collaborators

VTan

The Intellect Company

Investigators

Principal Investigator: Oliver Suendermann National University, Singapore

More Information

Go to

Responsible Party:
VTan, Co-Investigator, National University, Singapore

ClinicalTrials.gov Identifier:
NCT04911803 History of Changes

Other Study ID Numbers:
NUS-IRB-2021-266

First Posted:
June 3, 2021 Key Record Dates

Last Update Posted:
July 26, 2022

Last Verified:
July 2022

Individual Participant Data (IPD) Sharing Statement:

Plan to Share IPD:
No

Plan Description:

Research data will be stored in an encrypted NUS nbox folder with viewing-only permissions set and restricted to the PI and co-investigator. Primary data will be kept secured in electronic form by the PI and co-investigator in a format that precludes subsequent alteration. Research data files (e.g Excel sheets) will only be accessible by PI and Co-PI. Survey data will be collected on Qualtrics secured with an account password and 2FA verification. Upon completion of the data collection, personal identifiable information(e.g. matric number from RP pool students) will be removed from the Qualtrics file, and the MS Excelsheet containing the data will be encrypted and stored in an encrypted NUS nbox folder, accessible only by the PI and Co-investigator. App data will be linked to a participant code given to participants. However, no personal identifiable data will be collected through the wellbeing app programs. Personal data will be only be shared with PI and Co-I as mentioned above.

Studies a U.S. FDA-regulated Drug Product:

No

Studies a U.S. FDA-regulated Device Product:

No

Keywords provided by VTan, National University, Singapore:

Self-help
App
Wellbeing
Experimental
